# Supplementary figures and images for: Fezf2-positive fork cell-like neurons in the mouse insular cortex
Source: PLoS One. 2022 Sep 6;17(9):e0274170. doi: 10.1371/journal.pone.0274170 (PMC9447900; doi:10.1371/journal.pone.0274170)

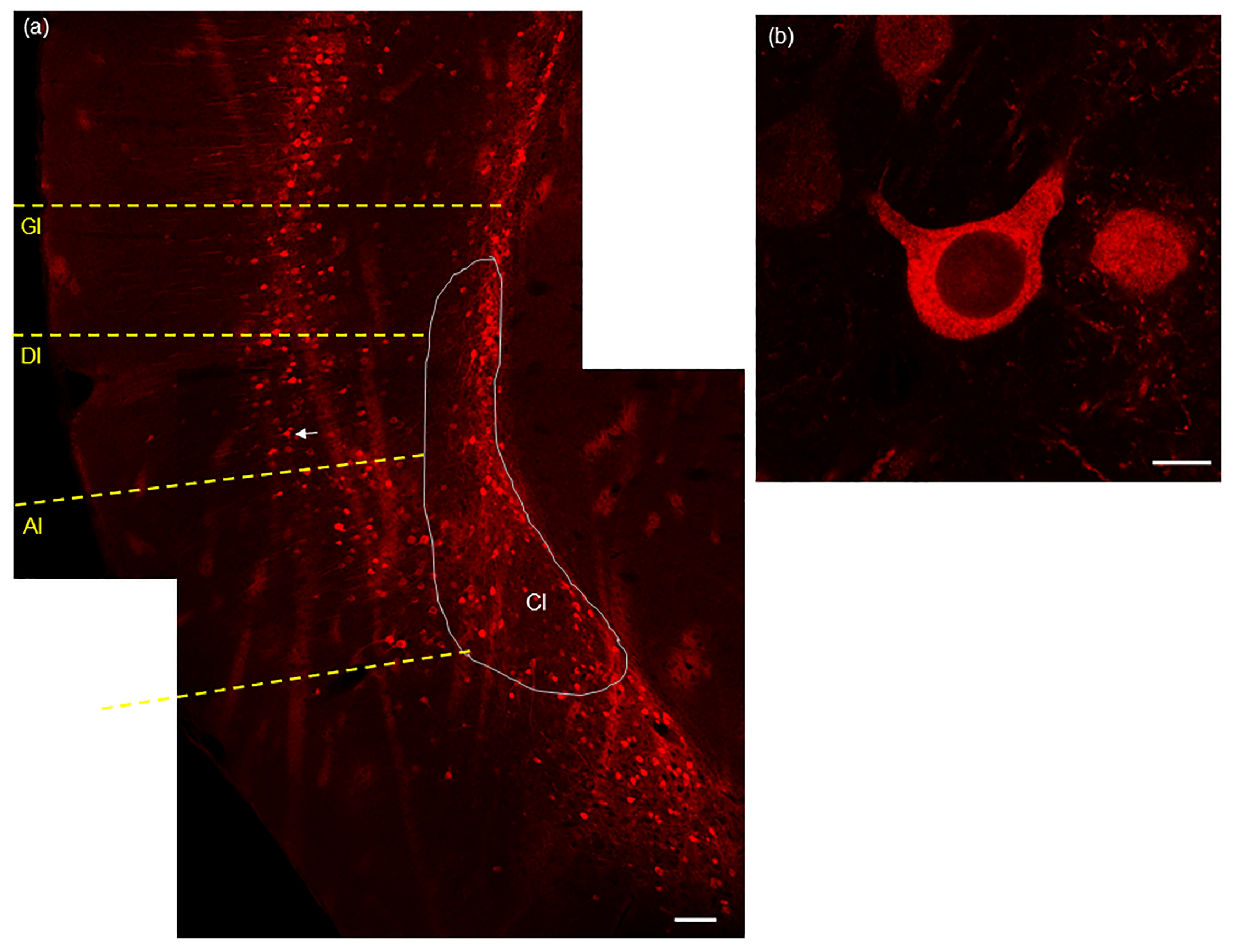

Supplement: S1 Fig — Some Fezf2-positive neurons in the mouse insular cortex exhibited fork cell-like shape. Immunofluorescence against RFP was performed to visualize Fezf2 signals with brain sections of Tg(Fezf2-tdTomato) mouse. (a) Fezf2 was expressed in layer Ⅴ and Ⅵ. (b) High-magnification image of a fork cell-like neuron indicated by the white arrow in the insular cortex of (a), which had two apical dendrites. Scale bars (a): 100 μm, (b) 10 μm. (TIF) [file pone.0274170.s001.tif]

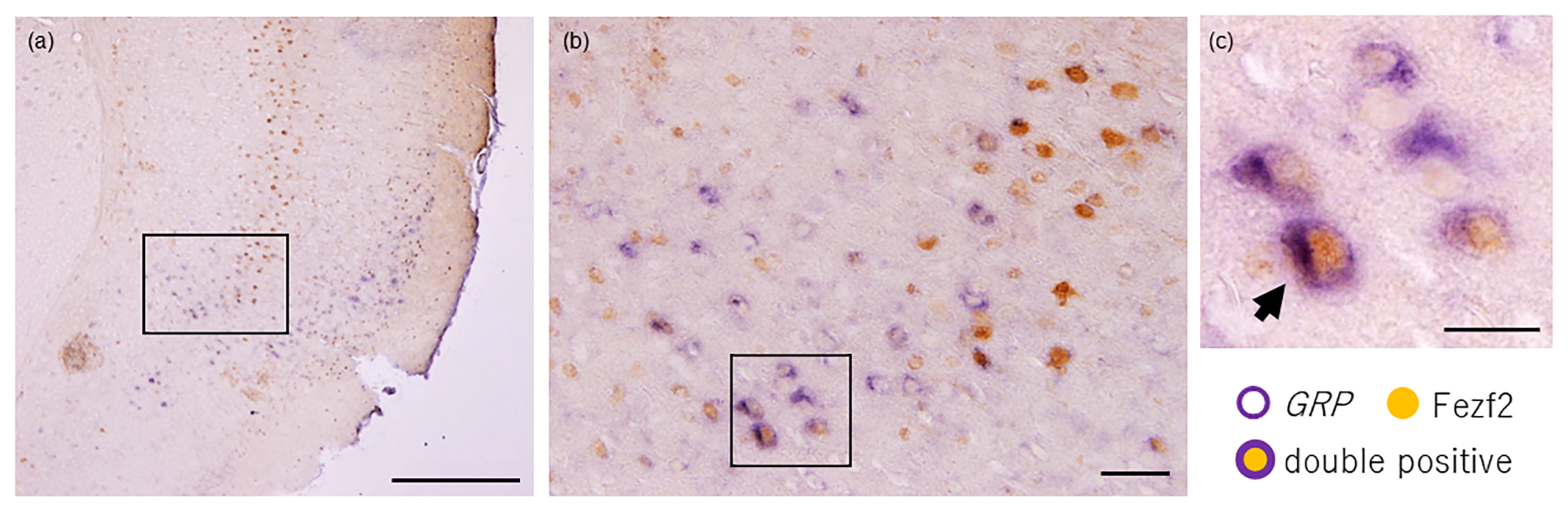

Supplement: S2 Fig — (a) IHC for RFP was performed after in situ hybridization against GRP. Tg(Fezf2-tdTomato) mouse. Scale bar: 200 μm. (b) A high-magnification image of (a). Scale bar: 50 μm. (c) A high-magnification image of (b). The black arrow indicates a neuron co-expressing GRP and Fezf2. Scale bar: 25 μm. (TIF) [file pone.0274170.s002.tif]
