# Supplementary material for: Fezf2-positive fork cell-like neurons in the mouse insular cortex
Source: PLoS One. 2022 Sep 6;17(9):e0274170. doi: 10.1371/journal.pone.0274170 (PMC9447900; doi:10.1371/journal.pone.0274170)
Supplement: Attachment — Submitted filename: Response to Reviewers.docx [file pone.0274170.s004.docx]

Dear Editor and Reviewers,

Thank you very much for reviewing our manuscript and offering valuable advises.

We had addressed your comments with point-by-point responses and revised the manuscript accordingly.

Responses to the Comments by the Academic editor:

1. *This academic editor think that, especially, more detailed morphological analysis is required as pointed out by Reviewer 2.*

Thank you very much for your invaluable comments.

According to your comments, to further investigate the fine structure of fork cell-like neurons, we reconstructed 3D fluorescent images of these neurons that were observed in the mouse insular cortex. With newly reconstructed 3D images, it is easy for us to observe a fork-cell like morphology (S1 Fig. and movie S1).

We would like to thank Reviewer #1 for his/her valuable comments on our manuscript.

Reviewer #1's comment:

Major
1. *At the regional level, authors showed the presence of neurons expressing Fezf2, neuromedin B mRNA, and gastrin-releasing peptide mRNA in the mouse insular cortex. However, it is still unclear whether or to which extents Fezf-positve neurons express these peptides. This can be answered by combining ISH for these peptides with immunohistochemistry for Fezf2 (RFP), and should strengthen the conclusion of this study.*

[Response to Reviewer #1]

Thank you very much for the favorable comments. According to your comments, we investigated whether *GRP/MNB* and Fezf2 were co-expressed in a neuron of the mouse insular cortex. *GRP* and Fezf2 were co-expressed in some neurons of the mouse insular cortex. We showed these results in the Supporting Information S2 Fig. We changed the text of the ‘Results’ sections as follows: Next, we investigated whether *GRP* and Fezf2 were co-expressed in some neurons of the mouse insular cortex. Once the ISHH signal detection against *GRP* was completed with frozen brain sections of Tg(Fezf2-tdTomato) mouse, we performed immunohistochemistry using antibody against RFP. As is shown in S2 Fig., *GRP* and Fezf2 were co-expressed in some neurons of the mouse insular cortex (S2 Fig (c)). (Page 16, Lines 245-249).

We also changed the text of the ‘Methods’ sections as follows: **ISHH-IHC dual staining method** Shortly after the ISHH signal detection was completed, the sections were treated in PBS and then processed for Fezf2 IHC staining as described above. (Page 10, Lines 161-163).

However, we **could not clearly confirm** whether *MNB* and Fezf2 were co-expressed because of high nonspecific signals. Therefore, we did not include these results in our revised manuscript.

2. *In method sections for Tissue Preparation, anesthetize mice were intracardially perfused by either 4% PFA or 0.05%GLA/4% PFA. If fresh frozen sections were used for ISH experiment, additional information on the preparation of fresh frozen section from anesthetized mice is necessary. Please confirm this point.*

[Response to Reviewer #1]

Fresh frozen sections were not used for ISHH (ISH) experiments.

Minor

3. *Reported cell maker molecules for human von Economo neurons and/or folk cells are listed in Table 1. Based on this, authors selected Fezf2 to examine expression profiles in the mouse insular cortex. It is better to be explained why Fezf2 is selected from the listed molecules.*

[Response to Reviewer #1]

Since Fezf2-tdTomato mouse gave us good enough signals for us to visualize the whole morphology of a neuron, including axons and dendrites, and to identify fork cell-like neurons, we used Fezf2-tdTomato mouse for the identification of von Economo neurons and/or folk cells or the equivalents. Therefore, in our revised manuscript, we changed the text of the ‘Results’ sections as follows: Since tdTomato expression is driven by Fezf2 promoter in Fezf2-tdTomato mouse, Fezf2-tdTomato mouse allowed us to visualize whole cell morphology including axons and dendrites comparable to Golgi staining. We used Fezf2-tdTomato mouse for the identification of von Economo neurons and/or folk cells. (Page 14, Lines 200-203)

We would like to thank Reviewer #2 for his/her valuable comments on our manuscript.

Reviewer #2’s comment:

Major
1. *The major concern would be the morphologies they demonstrated are not fully convincing. Neurons in Figures 2f, 3e and 4c appears to have two apical processes, but how the authors exclude the possible existence of additional major process. Currently researchers are able to capture 3D morphology of cells by using the software such as Imaris or something comparable. Alternatively a sequential scanning EM, which provide 3D images of EM, would be available to show more precise primary dendritic arbor.*

[Response to Reviewer #2]

Thank you very much for the comments.

According to your comments, we reconstructed 3D fluorescent images of neurons. Reconstructed 3D images clearly showed the existence of Fezf2-positive neurons with fork cell-like morphology (S1 Fig. and movie S1). We changed the text of the ‘Results’ sections as follows: Next, to consolidate our findings, we reconstructed 3D images of fork cell-like neurons that were observed in the mouse insular cortex. We processed Z-axis image stacks for reconstruction. Neurons that had two apical dendrites were clearly recognized (Movie S1). (Page 16, Lines 235-237).

We also changed the text of the ‘Methods’ sections as follows: **3D images** Confocal Z stack images were acquired using a Zeiss LSM 810 confocal microscope and Zen software (Carl Zeiss). These images were collected at 1024 × 1024 pixel resolution. For 3D rendering, images were collected at 8bit with the optimal slice settings. Compiled software of Zeiss microscope was used for image analysis and 3D rendering. (Page 11, line186- page 12, line190)

Major
2. *They concluded that the additional possible markers such as NMB, GRP, VMAT2, and GABRQ seem negative in FC, although they need more precise labeling indicating both the two apical processes and immunostaining simultaneously. If the authors found a specific combination of markers are useful for the identification of FC, it would be a good job.*

[Response to Reviewer #2]

According to the Reviewer’s comments, we investigated whether GRP/MNB and Fezf2 were co-expressed in some neurons of the mouse insular cortex. As a result, *GRP* and Fezf2 were co-localized in some neurons of the mouse insular cortex (S2 Fig.). We changed the text of the ‘Results’ sections as follows: Next, we investigated whether *GRP* and Fezf2 were co-expressed in some neurons of the mouse insular cortex. Once the ISHH signal detection against *GRP* was completed with frozen brain sections of Tg(Fezf2-tdTomato) mouse, we performed immunohistochemistry using antibody against RFP. As is shown in S2 Fig., *GRP* and Fezf2 were co-expressed in some neurons of the mouse insular cortex (S2 Fig (c)). (Page 16, Lines 245-249).

We also changed the text of the ‘Methods’ sections as follows: **ISHH-IHC dual staining method** Shortly after the ISHH signal detection was completed, the sections were treated in PBS and then processed for Fezf2 IHC staining as described above. (Page 10, Lines 161-163).

However, we **could not clearly confirm** whether *MNB* and Fezf2 were co-expressed because of high nonspecific signals. Therefore, we did not include these results in our revised manuscript.

Minor
*2. The authors used ICR strain of mice. Why do authors used this strain rather than C57BL6. This should be mentioned.*

[Response to Reviewer #2]

Because the genetic background of Fezf2-tdTomato mouse is ICR, we used this strain. In order to respond to the comments, we performed ISHH using C57BL6 strain of mice and had the same results. We did not mention these results in our revised manuscript.

Minor
3. *As for the holding neurons, how many cells are examined with the EM. Do all the FC cells have this type of cells between the two proximal dendrites.?*

[Response to Reviewer #2]

Six cells were examined by electron microscopy. Almost all fork cell-like neurons had the same type of dendrites.
